# Reprogramming Skin Aging: A Regenerative and Epigenetic Perspective on Cutaneous Longevity

**DOI:** 10.1111/jocd.70788

**Published:** 2026-03-06

**Authors:** Diala Haykal, Michael Gold, Edward Lain, Jeremy Green, Patricia K. Farris

**Affiliations:** ^1^ Centre Médical Laser Palaiseau Private Practice Palaiseau France; ^2^ Medical Director, Gold Skin Care Center, Tennessee Clinical Research Center Nashville USA; ^3^ Sanova Dermatology, Adjunct Clinical Professor, Texas A&M College of Medicine USA; ^4^ Skin Associates of South Florida Skin Research Institute Florida USA; ^5^ Tulane University School of Medicine Department of Dermatology Louisiana USA

**Keywords:** epigenetic clocks, longevity, peptides, regenerative dermatology, skin aging

## 1 Introduction

Aesthetic dermatology is undergoing a transformative shift, one that mirrors the broader societal focus on longevity and proactive health optimization. Traditionally, the goals of aesthetic medicine were tied to visible rejuvenation, smoothing wrinkles, restoring volume, and refining contours. Today, patients increasingly seek interventions that not only enhance appearance, but also preserve the vitality, structure, and biological performance of their skin over time.

This shift reflects the evolving science of longevity, which distinguishes between lifespan, the number of years a person lives, and healthspan, the number of years lived in good health, free from disease and functional decline. In dermatology, an analogous concept is emerging, skin healthspan, or skinspan, the duration over which the skin maintains optimal barrier function, immune defense, regenerative capacity, and aesthetic quality [1].

Cutaneous aging, like systemic aging, is now understood as a modifiable process, shaped by intrinsic genetic programs and extrinsic stressors such as UV exposure, pollution, and inflammation. Advances in epigenetics, cellular senescence research, and regenerative technologies offer an opportunity to shift the focus from late‐stage correction to early, proactive biological support [2]. This commentary explores mechanistic pathways underlying skin longevity, including telomeric preservation, epigenetic clocks, senescence reversal via partial reprogramming, and the modulation of mitochondrial function through biomimetic peptides and non‐ablative energy‐based technologies.

## 2 The Biology of Skin Aging: A Modifiable Process

Cutaneous aging, once thought to be an inevitable accumulation of damage, is now understood as a modifiable process governed by intrinsic molecular pathways and extrinsic environmental influences. Telomere shortening, mitochondrial decline, and epigenetic drift represent key intrinsic changes driving cellular senescence. While these processes have been predominantly studied in fibroblasts due to their experimental accessibility, they are believed to affect all skin cell populations, including keratinocytes and melanocytes. These intrinsic alterations collectively impair extracellular matrix homeostasis and epidermal renewal, contributing to global skin aging [3].

Emerging evidence from in vivo and in vitro studies indicates that partial cellular reprogramming, particularly through transient expression of Yamanaka factors or through defined mRNA‐based cocktails composed of short‐lived synthetic mRNAs encoding selected epigenetic modifiers, transcriptional regulators, and rejuvenation‐associated factors [4]. While full reprogramming induces pluripotency, partial approaches reset epigenetic marks, improve mitochondrial function, and restore youthful transcriptomic signatures. In the context of skin, fibroblasts and keratinocytes exhibit remarkable responsiveness to reprogramming stimuli, suggesting that the skin may serve as an ideal organ for testing rejuvenation paradigms [5]. Recent studies have shown that localized, controlled reprogramming can reduce senescence markers, enhance extracellular matrix remodeling, and potentially extend cellular healthspan [6, 7]. These insights pave the way for non‐invasive or minimally invasive interventions that mimic or trigger endogenous reprogramming pathways, whether via energy‐based devices (EBDs), topically delivered RNAs, or nanocarrier systems targeting epigenetic regulators [8].

Recent advances in biogerontology have identified twelve hallmarks of aging, including genomic instability, loss of proteostasis, cellular senescence, chronic inflammation, altered mechanical properties, disabled macroautophagy, among others, and epigenetic alterations among others [9]. These hallmarks are not only relevant to systemic aging but also manifest distinctly in the skin, where they drive visible and functional decline. Importantly, each hallmark is associated with measurable biomarkers, such as advanced glycation end products (AGEs), matrix metalloproteinase (MMP) activity, and senescence‐associated β‐galactosidase expression [10]. In dermatology, tracking these biomarkers opens the door to objective assessment of skin aging and responsiveness to intervention [11].

Epigenetic clocks based on DNA methylation patterns have emerged as precise biomarkers for biological age. Recent efforts have tailored these clocks to skin‐specific contexts, revealing their utility in capturing both intrinsic aging and cumulative environmental stress [12, 13, 14]. The development of such metrics enables clinicians to move beyond chronological age and assess the regenerative potential of skin in a more biologically meaningful way. This shift repositions the goal of dermatological intervention from cosmetic improvement to upstream modulation of aging pathways, laying the groundwork for integrative longevity care.

This scientific framing shifts the goal in aesthetic medicine: no longer to camouflage signs of aging but to act upstream, targeting the cellular and molecular dysfunctions that underlie cutaneous deterioration. This paradigm demands a new generation of tools, technological, molecular, and diagnostic, that go beyond symptom management and support true regenerative care.

## 3 Energy‐Based Devices and Epigenetic Rejuvenation

Non‐ablative EBDs, such as fractional lasers, microneedling radiofrequency, and photobiomodulation systems, have emerged as pivotal tools in regenerative dermatology. Unlike ablative devices, which thermally ablate the epidermis and dermis and require extended recovery, non‐ablative modalities act on deeper dermal structures while for the most part preserving the skin barrier. These devices trigger controlled dermal microinjury, which activates fibroblasts, stimulates angiogenesis, and enhances extracellular matrix remodeling [15].

Recent findings suggest that the biological effects of these technologies extend to epigenetic modulation [16]. As demonstrated in Haykal's study on laser‐induced epigenetic modulation, non‐ablative fractional lasers may influence skin regeneration by altering epigenetic pathways [8]. Additionally, controlled heat shock from radiofrequency‐based devices can upregulate sirtuin pathways and antioxidant defenses, thereby enhancing cellular resilience [17, 18].

The ability of EBDs to stimulate rejuvenation without cell replacement opens the door to therapeutic strategies that mimic partial reprogramming. Fibroblasts subjected to repetitive microthermal stimulation show increased collagen gene expression and decreased MMP activity, suggesting epigenetic remodeling toward a more youthful phenotype [8, 15]. When incorporated into long‐term preventive protocols, these devices may not only reverse early signs of aging but extend the functional lifespan of cutaneous architecture. Rather than treating aging reactively, clinicians can now envision protocols that sustain skin vitality in synchrony with the tissue's biological rhythm.

## 4 Bioactive Molecules in Regenerative Dermatology: Topical, Oral, and Injectable Strategies

A growing body of research supports the role of bioactive molecules in targeting the biological mechanisms underlying skin aging. Topical formulations containing biomimetic peptides, antioxidants, polyphenols, and DNA repair enzymes are designed to modulate pathways related to collagen synthesis, oxidative stress, and inflammation [19]. For instance, copper peptides such as GHK‐Cu have demonstrated the ability to stimulate TGF‐β signaling in dermal fibroblasts, enhancing procollagen expression while downregulating MMPs [20].

Peptides targeting IGF‐1R, FGFR, and integrins have shown potential in activating fibroblast proliferation and improving dermal elasticity. Advances in delivery systems, including liposomal encapsulation, nanocarriers, and exosome‐inspired vesicles, have further increased the bioavailability of these agents, enhancing their impact on the skin's deeper layers [21].

Injectable therapies have evolved from volumizing agents to regenerative biostimulators. Non‐crosslinked hyaluronic acid, polynucleotides, amino acid complexes, and biostimulatory substances like poly‐L‐lactic acid and calcium hydroxylapatite have demonstrated effects beyond physical support. These injectables interact with the dermal microenvironment, activating TLRs, promoting fibroblast migration, and modulating cytokine profiles. For example, polynucleotides upregulate VEGF expression and enhance tissue oxygenation, while poly‐L‐lactic acid has been linked to IL‐6‐mediated collagen remodeling [22].

Systemic interventions, such as oral collagen peptides, coenzyme Q10, ceramides, and antioxidants like astaxanthin and resveratrol, are also under investigation [23]. These agents may reduce systemic oxidative stress, modulate NF‐κB activation, and improve mitochondrial function in cutaneous cells. Although outcomes remain heterogeneous, the theoretical foundation for combining internal and external interventions, the “In & Out” approach, suggests a promising integrative pathway for cutaneous longevity.

Patient experience and perception are increasingly central to therapeutic success. Psychodermatological insights reveal that patients seek alignment between visible improvements and inner well‐being. This dual emphasis on “How do I look?” and “How do I feel?” encourages a holistic model of care, where regenerative interventions not only rejuvenate the skin but enhance psychological resilience and confidence.

## 5 The Skin Microbiome and Neurocutaneous Pathways in Longevity

The skin microbiome plays a crucial role in maintaining cutaneous homeostasis, immune modulation, and barrier function. Commensal organisms influence host gene expression through microbial metabolites such as short‐chain fatty acids and indole derivatives, which activate anti‐inflammatory pathways and promote epithelial repair. Dysbiosis, characterized by reduced microbial diversity or overgrowth of pathobionts, has been linked to barrier dysfunction, accelerated aging, and inflammatory dermatoses [24].

Cutaneous aging is also modulated by neurocutaneous interactions. The skin‐brain axis mediates stress responses via corticotropin‐releasing hormone (CRH), ACTH, and cortisol, all of which impact dermal repair, antioxidant capacity, and collagen integrity [25]. Chronic psychological stress elevates cortisol levels, which downregulates Nrf2‐mediated antioxidant defenses and upregulates MMP expression, leading to tissue breakdown [26].

In parallel, gut microbial communities exert systemic effects on skin physiology. Gut dysbiosis, characterized by reduced microbial diversity or overgrowth of pro‐inflammatory strains, has been linked to chronic low‐grade inflammation and oxidative stress. This is mediated in part by short‐chain fatty acids (SCFAs) such as acetate and propionate, which can translocate systemically and modulate redox balance positively by activating the Nrf2 pathway. SCFAs exert anti‐inflammatory and antioxidant effects, contributing to cutaneous resilience when present in a balanced microbial environment [27]. Clinical associations observed in rosacea and atopic dermatitis highlight the relevance of this gut‐skin axis in inflammatory and age‐related skin conditions [28, 29, 30, 31]. Accordingly, therapeutic interventions aimed at restoring gut microbial homeostasis, through targeted nutrition, prebiotics, probiotics, or postbiotics, may represent a valuable adjunct in protocols targeting skin longevity and resilience [24].

Emerging neurocosmetic strategies, such as topicals targeting neuromediators or protocols incorporating circadian regulation and sleep hygiene, aim to reduce stress‐induced skin damage [32]. These findings underscore a biopsychosocial model of skin aging, where neurohormonal balance, microbial diversity, and cellular resilience are interdependent domains. Addressing these layers in concert may offer the most durable protection against biological aging.

## 6 Toward a Skin Longevity Index: A New Clinical Metric

**FIGURE 1:**
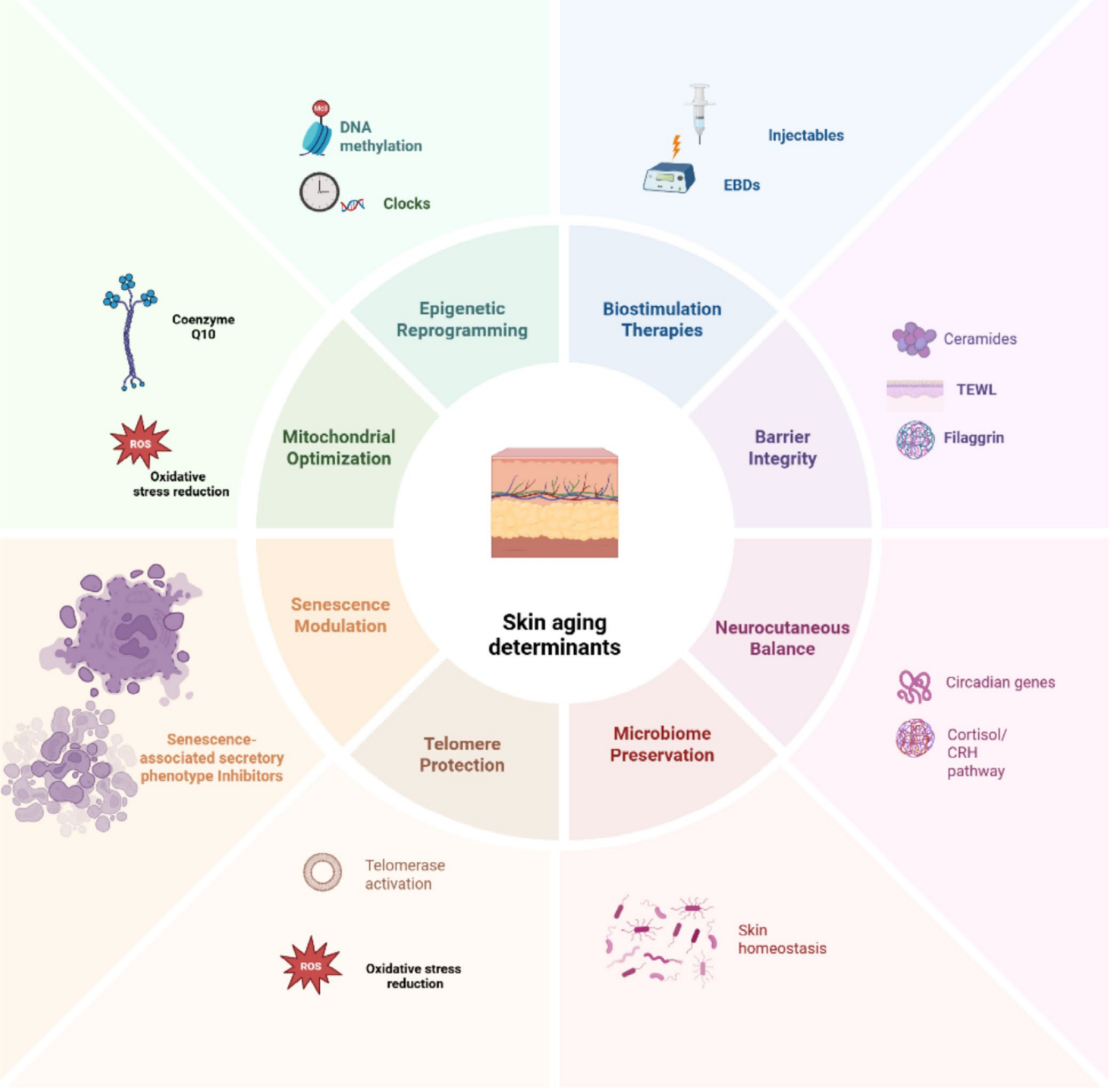
Skin Longevity Index: Multidimensional Determinants and Biomarkers.

Despite advances in aesthetic tools, the field still lacks a standardized, biologically relevant metric to assess skin aging and regenerative progress. Current visual or tactile scales provide limited insight into underlying cellular or molecular health. We propose the development of a Skin Longevity Index (SLI), a composite metric incorporating structural, functional, and biochemical parameters to assess regenerative capacity (Figure 1).

Such an index could incorporate quantitative measures of transepidermal water loss (TEWL), collagen and elastin density assessed via non‐invasive imaging, senescence‐associated β‐galactosidase activity, skin‐specific DNA methylation age, epidermal turnover rates, and the expression profiles of core circadian genes such as BMAL1 and PER2 [33, 34, 35]. These parameters, when integrated, could provide a multidimensional profile of cutaneous aging, enabling clinicians to stratify patients, personalize treatments, and track longitudinal responses to regenerative protocols.

Beyond clinical utility, the SLI could serve as a research tool for evaluating interventions in controlled trials, allowing comparison across modalities and supporting evidence‐based innovation. Its development will require interdisciplinary collaboration among dermatologists, bioinformaticians, epigeneticists, and systems biologists.

To facilitate clinical adoption, we propose implementing the SLI entirely through non‐invasive modalities. Parameters such as DNA methylation and transcriptomic signatures can be collected via tape stripping, while dermal collagen and elastin density are measured by optical coherence tomography or high‐frequency ultrasound. Barrier function is assessed by TEWLometry, and circadian gene expression patterns can also be derived from surface sampling. A four‐step workflow, initial bedside assessment, algorithmic scoring, personalized interventions, and longitudinal reassessment, could integrate seamlessly into dermatological practice. This non‐invasive approach eliminates the need for skin biopsies, aligning the SLI with real‐world feasibility and patient comfort.

## 7 Discussion

The convergence of regenerative biology, molecular dermatology, and psychosocial science is reshaping the goals of aesthetic medicine. No longer limited to surface corrections, the field is positioned to influence aging at its biological roots. This shift necessitates a rethinking of education, clinical protocols, and research frameworks. Training programs should integrate principles of aging biology, epigenetics, and systemic resilience, while emerging technologies, ranging from wearable biosensors to AI‐driven diagnostics, should be embraced to personalize patient care.

Preclinical studies using murine models and human skin organoids can validate hypotheses about reprogramming, senescence modulation, and mitochondrial rejuvenation. Organoid platforms derived from patient iPSCs offer exciting potential to assess intervention efficacy in vitro while maintaining patient‐specific cellular signatures. Combined with in vivo assessments of epigenetic age and molecular biomarkers, these tools could drive precision in both research and clinical translation.

Patients are already seeking treatments that merge aesthetic and health outcomes. Aesthetic dermatologists are uniquely positioned to respond to this demand by integrating mechanistic insight with patient‐centered care. By doing so, they can lead the transformation of aesthetic medicine into a preventive, regenerative discipline grounded in the science of longevity.

## 8 Conclusion

Skin longevity is no longer a conceptual aspiration; it is an emerging, biologically grounded objective that bridges regenerative science and aesthetic practice. By harnessing the power of non‐ablative devices, biomimetic molecules, microbiome preservation, and neurocutaneous balance, clinicians can shift from correction to prevention, and from surface‐level beauty to deep, functional health.

The development of a Skin Longevity Index, alongside innovations in diagnostics and therapeutic personalization, offers a roadmap for future practice. As regenerative dermatology continues to evolve, it holds the promise not only of aesthetic enhancement, but of true biological rejuvenation.

## Author Contributions

D.H. drafted the manuscript. M.G., E.L., J.G., and P.K.F. reviewed the content and provided their feedback. All authors approved the final version of the manuscript.

## Funding

The authors have nothing to report.

## Ethics Statement

The authors have nothing to report.

## Conflicts of Interest

The authors declare no conflicts of interest.

## Data Availability

Data sharing not applicable to this article as no datasets were generated or analysed during the current study.
